# The Role of Inflammation in Neurodegenerative Diseases: Parkinson’s Disease, Alzheimer’s Disease, and Multiple Sclerosis

**DOI:** 10.3390/ijms26115177

**Published:** 2025-05-28

**Authors:** Justyna Fołta, Zuzanna Rzepka, Dorota Wrześniok

**Affiliations:** Department of Pharmaceutical Chemistry, Faculty of Pharmaceutical Sciences in Sosnowiec, Medical University of Silesia, Jagiellońska 4, 41-200 Sosnowiec, Poland; d201205@365.sum.edu.pl (J.F.); zrzepka@sum.edu.pl (Z.R.)

**Keywords:** inflammatory, neurodegenerative disease, central nervous system

## Abstract

Neurodegenerative diseases are a group of conditions that have in common the progressive damage and degeneration of neurons in the central nervous system. This group includes Parkinson’s disease, Alzheimer’s disease, and multiple sclerosis, among others. In recent years, increasing evidence has pointed to the important role of inflammation in the pathogenesis of these conditions. The occurrence of inflammation in the brain, which is often triggered by pro-inflammatory activation of microglia or astrocytes, can consequently lead to a chronic inflammatory response that contributes to the development of neurodegenerative processes. Inflammatory processes themselves, both within the nervous system and throughout the human body, appear to be central to the initiation and progression of neuronal damage. Understanding the role of inflammation in these diseases may open up new perspectives and opportunities in the future in the development of effective therapies to improve patients’ quality of life as the vast majority of cases of patients affected by neurodegenerative diseases continue to be treated symptomatically since causal treatments are lacking. In this review, we provide information on the impact of inflammation on the pathogenesis, course, and potential therapeutic options for selected neurodegenerative diseases. In addition, this article provides a general description of neuroinflammation and the involvement and role of specific cells in the central nervous system.

## 1. Introduction

The increase in life expectancy is accompanied by an increase in various types of disease, including neurodegenerative diseases within the central nervous system (CNS) [1,2]. Globally, it is estimated that the number of patients affected by dementia will triple in 2050 compared to 2019, reaching more than 150 million cases [3]. Neurodegenerative diseases include, for example, Parkinson’s disease (PD), Alzheimer’s disease (AD), and multiple sclerosis, which are characterized by progressive and selective neuronal loss, resulting in deterioration of the patient’s sensory–motor, cognitive, and emotional functions [4,5,6]. Although these diseases differ in their clinical manifestations, which depend on, among other things, what part of the brain is involved in the degenerative process, they share certain pathological features and characteristics. Several factors, such as genetic, environmental, and endogenous influences, contribute to the development of neurodegenerative diseases. Common pathophysiological mechanisms include neuroinflammatory processes [7,8], oxidative stress with reactive oxygen species [9,10,11], mitochondrial dysfunction [12,13], abnormal protein dynamics [14,15], DNA damage [16,17], and neurotrophin dysfunction [18,19]. Neuroinflammation serves as a protective response, initially safeguarding the brain by eliminating or inhibiting various pathogens [20,21]. Furthermore, dysregulated calcium signaling, which is a hallmark of neurodegeneration, plays a critical role in the pathogenesis of Alzheimer’s disease and other neurodegenerative disorders, contributing to neuronal dysfunction and cell death [22].

Neuroinflammation refers to an inflammatory response occurring within the central nervous system and it is present in a variety of neurological conditions, such as infectious, autoimmune, non-infectious inflammatory, demyelinating, and neurodegenerative diseases [23]. “Neuroinflammation” describes an inflammatory response within the central nervous system (CNS), marked by the accumulation of glial cells—especially astrocytes and microglia—as a reaction to injury [24,25]. Under conditions of cellular homeostasis, the immune response in the central nervous system is extinguished by appropriate cells, including microglia cells, the natural immune cells of the central nervous system, involved in neuronal development by phagocytosing and removing damaged neurons and synapses [26]. When a pathogen enters the CNS, innate immune cells are activated to develop innate immune responses, which then result in the recruitment of adaptive immune cells from the periphery. If the inflammatory response is sustained and hyper-reactive, nerve cell damage and neurodegenerative phenomena can result [27,28,29]. This review presents the role of inflammation occurring in the central nervous system in the pathogenesis and course of selected neurodegenerative diseases—Parkinson’s disease, Alzheimer’s disease, and multiple sclerosis—taking into account available studies and case reports. The aim was to approach the issue of the role of inflammation and neuroinflammation in neurodegenerative diseases in a systemic and functional way—our review highlights the importance of inflammation as a clinical phenomenon and potential therapeutic target.

## 2. The Role of Microglia and Astrocytes in the Inflammatory Process

The mechanisms underlying neurodegenerative diseases remain incompletely understood. Microglia and astrocytes are structures considered to be key factors in maintaining homeostasis within the central nervous system in the event of neuropathological conditions. Both microglia and astrocytes play a key role in regulating inflammatory responses in the central nervous system. These cells communicate with each other when an inflammatory response occurs [30,31].

Microglial cells are highly specialized cells found in the central nervous system, i.e., the brain and spinal cord. Microglial cells are a type of specialized macrophages and are non-neuronal cells of the central nervous system. In terms of numbers, they constitute about 10% of all cells of the central nervous system. Microglial cells arise from primitive yolk follicle macrophage precursors and their migration to the brain begins at an early stage of embryonic development, before the formation of the blood–brain barrier (BBB), where they remain until the BBB is fully formed. Microglia have self-repair capabilities and can regenerate, maintaining their population throughout life [32,33]. In pathological situations when the BBB is damaged, peripheral macrophages—which are not located in the brain—can infiltrate and contribute to inflammation. Microglial cells are classified as primary cells of the immune system, thanks to which they can detect endogenous and exogenous pathogens and influence the inflammatory process in two ways [34,35,36]. In response to tissue damage, infection, or other stressors, microglial cells are activated. During this process, they increase in size and begin to secrete pro-inflammatory cytokines, chemokines, and other signaling molecules such as cytokines or nitric oxide (NO) [32]. Activated microglial cells are polarized toward two phenotypes: M1 or M2. The M1 phenotype, a pro-inflammatory phenotype, is caused by cytokines such as interferon-α (IFN-α) and tumor necrosis factor-α (TNF-α), while the M2 phenotype, also known as the anti-inflammatory phenotype, is triggered by various cytokines such as interleukins (IL), including IL-4, IL-13, and IL-25. Chronic and long-term activation of microglia in response to chronic stress or pathological changes can lead to neuronal damage and increased inflammation, which is characteristic of many neurodegenerative diseases such as Alzheimer’s disease, Parkinson’s disease, and multiple sclerosis [21,37].

**Figure 1:**
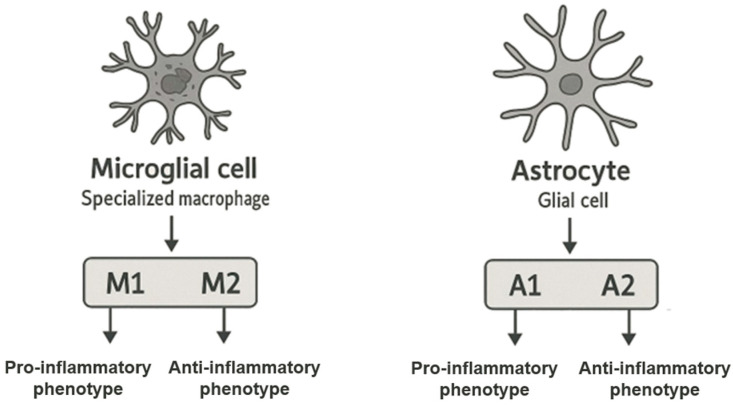
Phenotypic polarization of microglia and astrocytes in an inflammatory response.

Astrocytes are among the most numerous cells in the central nervous system, and their number is almost equal to the number of neurons. These are glial cells with a characteristic star shape that participate in many physiological processes in the brain and spinal cord. One of their basic roles is to create the blood–brain barrier [33]. Due to their presence, astrocytes allow the brain to maintain homeostasis, protecting it from harmful substances in the blood, while enabling the exchange of essential substances such as oxygen and glucose [38]. Astrocytes also play a key role in the process of synaptogenesis, or the process of creating new synapses. Importantly, this process occurs not only during the development of the central nervous system but also in response to its damage. Astrocytes participate in the repair and reorganization of neuronal networks in pathological situations such as injuries to the central nervous system or in the event of neurodegenerative diseases, which is necessary to restore the proper functions of damaged areas of the brain [39,40]. In addition, astrocytes respond to pro-inflammatory cytokines secreted by various cells, including microglia and peripheral immune cells recruited to the central nervous system. In response to these signals, astrocytes modulate inflammatory responses by influencing neighboring cells and changing the microenvironment in the brain. As a result, astrocytes can activate inflammatory responses to protect against pathogens but when chronically activated, they can contribute to neurodegeneration. Like microglia, astrocytes can adopt different phenotypes in response to inflammatory stimuli: a pro-inflammatory phenotype, A1, which promotes inflammatory processes and cell damage; and an anti-inflammatory phenotype, A2, which promotes regeneration and protection of neurons. Increased expression of glial fibrillary acidic protein is often used as a marker of astrocyte activation, allowing us to monitor their status in various pathological processes. All these functions make astrocytes play an extremely important role in maintaining balance in the central nervous system, both under physiological conditions and in response to various pro-inflammatory and stress-inducing factors and damage [41,42,43,44,45]. The phenotypic polarization of microglia and astrocytes in an inflammatory response is shown in Figure 1.

## 3. Parkinson’s Disease

### 3.1. Background Information

Parkinson’s disease (PD) is the second most common neurodegenerative disease, and its prevalence worldwide is currently around 6 million people. Over the past generation, the incidence of Parkinson’s disease has increased significantly, with the number of affected individuals rising by a factor of 2.5. This has made it one of the primary contributors to neurological disability, impacting not only the quality of life of individuals but also placing a significant burden on healthcare systems and caregivers [46,47]. The disease was first formally described in 1817 by Dr. James Parkinson in his publication “*An Essay on the Shaking Palsy*”, where he provided a detailed account of the symptoms and motor signs associated with the condition, which, in turn, led to the patient’s eventual death [48,49]. Parkinson’s disease is a progressive disease that is caused by the degeneration and loss of dopaminergic neurons in the substantia nigra, a key region of the brain involved in motor control. Characteristic symptoms of the disease include resting tremor, bradykinesia, i.e., the slowing down of movements, as well as hypokinesia, i.e., the low amplitude of movement. Moreover, patients frequently experience postural instability, which increases their risk of falling and causes challenges with balance. These motor issues are often paired with non-motor symptoms, including sleep problems, cognitive impairment, depression, and autonomic dysfunction, which further complicates both the diagnosis and treatment of the condition. Currently, Parkinson’s disease is described as a multisystem neurodegenerative disease because it simultaneously involves—in addition to the central nervous system (CNS)—the enteric nervous system, autonomic nervous system, immune system, and digestive tract [50,51,52]. In addition, the immune system is believed to play an important role in the pathogenesis and course of the disease. Microglia cells—or resident cells of the immune system in the brain—when dopaminergic neurons are lost, become activated, causing chronic inflammation within the central nervous system and neuronal damage. This phenomenon can, consequently, contribute to the accelerated degeneration of brain tissue and contribute to the appearance of motor and non-motor symptoms [53].

### 3.2. Pathogenesis

Parkinson’s disease is an age-related neurodegenerative disorder, with its incidence and prevalence steadily increasing with age. To date, the pathogenesis and causes of this disease have not been fully discovered. A characteristic feature of Parkinson’s disease is the gradual and progressive loss of dopaminergic neurons in the substantia nigra pars compacta, leading to a reduced amount of dopamine in the striatum [54,55].

One of the most important molecules involved in the pathogenesis of Parkinson’s disease is α-synuclein, which is a neuronal protein 140 amino acids long that is abundant in presynaptic nerve endings. This protein is found physiologically in the central nervous system, performing regulatory functions in the release of neurotransmitters and is responsible for maintaining neuronal plasticity; however, in the course of Parkinson’s disease, abnormal aggregation of α-synuclein protein results in the formation of deposits of so-called Lewy bodies, which are insoluble. This process correlates and is associated with the death of dopaminergic neurons and a consequent decrease in dopaminergic transmission [56,57]. α-Synuclein is synthesized in the peripheral nervous system and central nervous system, as well as in some non-neuronal tissues such as exocrine glands and hematopoietic tissues, areas where immune challenge is most likely to occur [58]. The research indicates that in the early stages of Parkinson’s disease, α-synuclein plays an important role in the interaction between neuronal dysfunction and the immune system, involving pro-inflammatory factors and the gut microbiota. The first premorbid pathological changes in α-synuclein in Parkinson’s disease are observed in the vagus nerve and the olfactory bulb. Post-mortem studies have also identified α-synuclein aggregates in the myenteric and submucosal plexuses of the enteric nervous system. Additionally, the nasal mucosa and swallowed mucus have been discussed as potential routes of pathological α-synuclein entry into the body. This indicates that pathological changes in α-synuclein protein may have their initiation in the intestines and then penetrate the central nervous system, suggesting that the intestines may be the initial source and pathway for the spread of the disease [59,60]. α-synuclein with pathological structures, i.e., mutated or in the form of aggregates, causes conformation-dependent strong inflammatory activation of human monocytes and BV2 microglia cells, with early mutations and aggregation of α-synuclein having the strongest effect on the immune response. Activation of the aforementioned cells by the protein in question is enhanced by extracellular vesicles, facilitating its uptake. Extracellular vesicles from the blood of patients with Parkinson’s disease induce stronger activation of monocytes than those from healthy individuals. Monocytes from patients with Parkinson’s overreact in response to abnormal α-synuclein. Moreover, it was noted in a mouse model that deformed α-synuclein in the central nervous system is sufficient to induce changes in the organization of monocytes within it [61]. It was also discovered that α-synuclein in vitro stimulates microglia cells to induce pro-IL-1β expression to a degree comparable to that observed after stimulation with the known inflammatory inducer lipopolysaccharide. In contrast, the step required for IL-1β release is phagocytosis by monocytes of α-synuclein F (fibrillar form) [62]. The very process of phagocytosis of microglia α-synuclein F and the release of cytokines is dependent on the inflammatory environment [63]. In the pathogenesis of Parkinson’s disease, a key role is played by α-synuclein, which activates both innate and adaptive immune responses through microglia, an increase in inflammatory cytokines, and an influx of T cells into the CNS. Studies have shown that peripheral CD4 and CD8 T cells in patients with Parkinson’s produce Th1/Th2 cytokines in response to α-synuclein, suggesting a chronic memory T-cell response. Studies in mice overexpressing α-synuclein showed infiltration of CD4 and CD8 cells producing interferon-γ. Removal of receptors for T cells, CD4, or the use of fingolimod (a compound that inhibits the migration of T cells from lymph nodes into the CNS) reduced the myeloid response and protected against the loss of dopaminergic cells, indicating the potential of targeted therapy in modifying Parkinson’s disease [64].

In the context of inflammation in the pathogenesis of Parkinson’s disease, the relationship of the gut microbiota to the incidence and pathogenesis of the condition is noteworthy. Two main mechanisms associated with the gut microbiota in the context of Parkinson’s disease have been postulated as follows:

1. A reduction in the number of short-chain fatty acid (SCFA)-producing bacteria and an increase in the number of mucin-degrading bacteria such as Akkermansia may lead to increased intestinal permeability, thereby allowing toxins such as lipopolysaccharides—a known pro-inflammatory factor—to access the nervous system and promote α-synuclein aggregation;
2. Deficiency of SCFA-producing bacteria can increase inflammation in the central nervous system by activating microglia [65].

Experimental studies using prebiotics have shown that fermentation of small fecal matter in patients with Parkinson’s disease increases SCFA production and alters the gut microbiota, suggesting that the patients’ microbiota responds positively to prebiotics. In an intervention study involving patients with newly diagnosed (*n* = 10) and treated Parkinson’s disease (*n* = 10), 10-day prebiotic therapy was associated with favorable changes in microbiota, SCFA production, inflammation, and neurofilament levels [66].

In the pathogenesis and course of Parkinson’s disease (PD), nigrostriatal dopamine neurons in the substantia nigra pars degenerate, resulting in a deficiency of the neurotransmitter dopamine in nerve endings in the striatum. Increased levels of pro-inflammatory cytokines, such as tumor necrosis factor-α, interleukin IL-1β, and IL-6, and decreased levels of neurotrophins, such as brain-derived neurotrophic factor, have been observed post-mortem or in the cerebrospinal fluid of affected patients. Abnormal levels of cytokines and neurotrophins can be initiated by activated microglia, which can then promote apoptotic cell death and subsequent phagocytosis of dopaminergic neurons [67].

Microglia cells themselves, as immune cells in response to various stimuli or pathogens, produce factors involved in the pathophysiology of Parkinson’s disease. These factors include tumor necrosis factor-α, interleukins (IL1-β, IL-6, IL-2, or IL-4), interferon-γ, prostaglandins (E2 and J2), reactive nitrogen species (nitric oxide and peroxynitrite), and reactive oxygen species (peroxides, hydrogen peroxide, or hydroxyl radicals) [68].

### 3.3. Treatment

The treatment of Parkinson’s disease by current treatment standards is symptomatic treatment as there is currently no medication to treat the disease causally. Pharmacological treatments include the use of decarboxylase inhibitor levodopa, dopamine agonists, monoamine oxidase-B inhibitors, catechol-O-methyltransferase inhibitors, amantadine, or anticholinergic drugs [69,70].

Non-steroidal anti-inflammatory drugs (NSAIDs) are widely used clinically to eliminate inflammation, pain, swelling, and stiffness in the limbs. Meta-analyses indicate that the use of non-aspirin anti-inflammatory drugs was significantly associated with a reduced risk of Parkinson’s disease [71]. In the course of Parkinson’s disease, there is an increase in neuroinflammatory reactions, which correlate positively with an increase in cyclooxygenase levels. In the course of the pathogenesis of this disease, increased expression of the mentioned enzyme and elevated levels of prostaglandin E2 are observed. Belonging to the group of non-steroidal anti-inflammatory drugs, ibuprofen is commonly used clinically in patients diagnosed with Parkinson’s disease to alleviate these inflammatory processes [72].

Minocycline, categorized as a semi-synthetic tetracycline, exhibits neuroprotective properties by inhibiting the key apoptosis-inducing enzymes caspase 1 and inducible nitric oxide synthase in animal models of parkinsonism; moreover, it reduces microglia activation induced by 6-hydroxydopamine and 1-methyl-4-phenyl-1,2,3,6-tetrahydropyridine, reducing the secretion of cytokines, chemokines, and inflammatory mediators. In addition, it prevents the loss of dopamine in the striatum and inhibits the migration of T lymphocytes, promoting the protection of nigrostriatal dopaminergic neurons [73,74,75].

**Figure 2:**
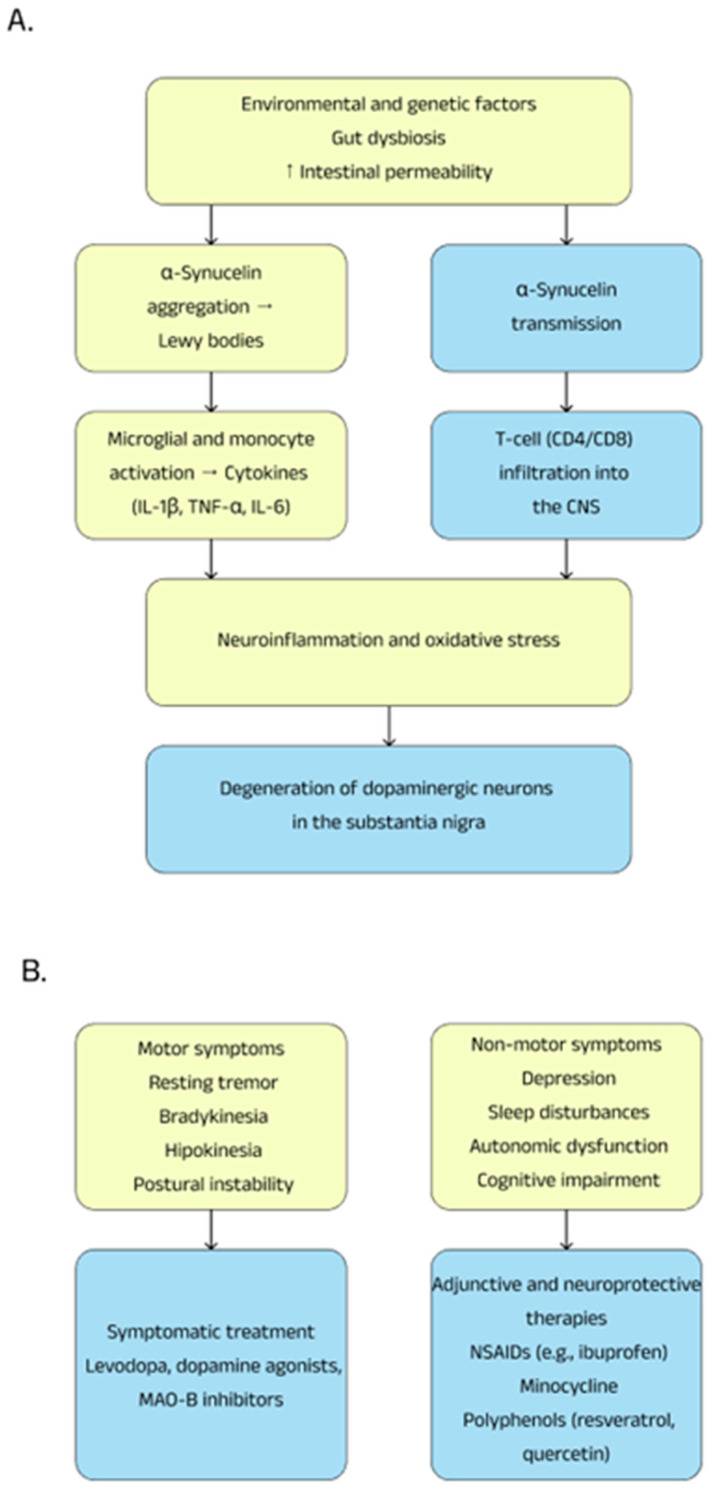
Pathogenesis (**A**) and therapeutic possibilities (**B**) in Parkinson’s disease.

Polyphenols play an important role in protecting against various diseases, such as cancer, diabetes, cardiovascular disease, and neurodegenerative diseases thanks to their ability to mitigate pathobiological processes. Research has shown that they can modulate the nuclear factor kappa-light-chain-enhancer signaling pathway of activated B cells, contributing to the inhibition of neuroinflammation. In vivo and in vitro experiments suggest that compounds such as resveratrol, proanthocyanidin, and quercetin affect various pathological mechanisms, helping to maintain oxidative balance and reducing neuroinflammatory processes, which may have beneficial effects for protection against Parkinson’s disease. Studies have shown that, contained in wine and grape skins, resveratrol exerts a protective effect on dopaminergic neurons by preventing lipopolysaccharide-induced neurotoxicity in a dose- and time-dependent manner. This mechanism involves inhibiting microglial activation, which subsequently reduces the release of pro-inflammatory factors [76,77]. Summary information on Parkinson’s disease is presented in Figure 2.

## 4. Alzheimer’s Disease

### 4.1. Background Information

Alzheimer’s disease (AD), classified as a dementia disorder, is the most common neurodegenerative disease and is a growing public health problem worldwide. Every three seconds, someone in the world develops dementia, with an estimated 55 million people affected in 2019. Projections by the World Health Organization (WHO) indicate that this number could rise to 139 million by 2050 [78,79]. Alzheimer’s disease was first described in 1907 by German psychiatrist and neuropathologist Alois Alzheimer. He identified the case of a 51-year-old woman, Auguste Deter, who suffered from progressive memory loss, disorientation, and behavioral changes that were disturbing to those closest to her. After her death, changes characteristic of the disease were observed in her brain, such as amyloid plaques and neurofibrillary tangles. Since then, that is, for almost more than 120 years, numerous attempts have been and are being made to understand the pathology of the disease and to develop diagnostic and therapeutic tools. The breakthrough came only at the end of the 20th century, when the details of the pathological features of the disease were determined and its genetic subtypes were identified [80,81,82]. Alzheimer’s disease is not homogeneous; it progresses slightly differently in each patient and can affect cognitive function, leading to gradual memory loss, and difficulty with thinking, language, and problem-solving. In most patients, symptoms also include mood disorders, confusion, and gradual loss of contact with the environment [83,84,85]. Alzheimer’s disease can develop over a long period of time, and the brain is able to maintain homeostasis and stability for about 20 years before cognitive decline begins to occur in the middle stage of the disease [86].

Alzheimer’s disease is a multifactorial condition, and the two main hypotheses for its causes are the cholinergic and amyloid hypotheses. The cholinergic hypothesis assumes that a loss of cholinergic function contributes to cognitive decline in neurodegenerative diseases, which include the disease in question. Abnormalities in the cholinergic system can lead to abnormal phosphorylation of the tau protein, inflammation, neuronal apoptosis, and other pathological events. The second hypothesis is the amyloid hypothesis, assuming that the main cause of Alzheimer’s disease is the accumulation of amyloid (Aβ) plaques in the brain, which result from a disturbed Aβ clearance mechanism. Aβ plaques induce neuroinflammation, damage nerve cells, and activate a pathological cascade involving tau phosphorylation and neurodegeneration [87,88,89]. Alzheimer’s disease is associated with a variety of risk factors that can influence its development. Age is one of the main determinants, with a marked increase in risk with aging. In addition, genetic factors play a key role, contributing to 60–80% of cases of the disease. The prevalence of AD in women is higher than in men, who are at a 19–29% lower risk of developing the disease. Also, inflammation in the central nervous system, which can accelerate degenerative processes, plays an important role in the pathogenesis of AD [84,90,91]. Interestingly, in studies performed on mice, microglia extracted from healthy males show a higher expression of inflammatory markers, while microglia extracted from female individuals have a more neuroprotective profile. However, with advancing age, this pattern changes—females show stronger activation of microglia in the brain, which, compared to males, may increase the risk of developing Alzheimer’s disease [92].

### 4.2. Pathogenesis

The pathogenesis of neurodegenerative Alzheimer’s disease is a complex process, dependent on many factors, and is associated with the accumulation of extracellular amyloid β (Aβ) deposits and the formation of intracellular neurofibrillary tangles—composed of hyperphosphorylated τ protein—in cortical and limbic areas of the human brain. Another important mechanism for the onset of Alzheimer’s disease is the activation of microglia cells, which are responsible for inflammatory reactions within the central nervous system. The onset of chronic inflammation in the brain intensifies neurodegenerative processes, thereby exacerbating the progression of the disease [93,94].

Numerous cell types and molecules such as astrocytes, microglia, cytokines, and chemokines are involved in the neuroinflammatory process associated with Alzheimer’s disease, which consequently leads to negative effects on surrounding neurons, causing oxidative stress and apoptosis, among other things [95]. A meta-analysis of studies of inflammatory markers showed that elderly patients with Alzheimer’s disease had elevated levels of peripheral blood interleukin-1β [96].

Depending on the stage of Alzheimer’s disease and co-modifying factors, microglia can exhibit dual effects of both protective and damaging neurons. In the early stages of Alzheimer’s disease, activated microglia cells perform protective functions for surrounding neurons and synapses, removing β-amyloid plaques by phagocytosis, also releasing proteases and engulfing dead cells. In advanced stages of the disease, Aβ removal and tau accumulation are impaired, which leads to microglia dysfunction, causing the overexpression of pro-inflammatory cytokines, and consequently damaging astrocytes and neurons [97]. An important pathological mechanism in Alzheimer’s disease is cytokine production, which plays a key role in the progression of inflammatory processes. Analysis performed in a mouse model shows a significant increase in the levels of pro-inflammatory cytokines, such as TNF-α, IL-6, IL-12p40, IL-1β, IL-1α, and GM-CSF, compared to healthy normal mice. The results suggest that an enhanced inflammatory response may be important in the pathogenesis of AD and be related to the presence and aggregation of Aβ [98]. Microglia activation plays an important context- and stage-dependent role in Alzheimer’s disease progression. Damaged neurons release signals that induce excessive activation of microglia, leading to cyclic neuronal damage known as reactive microglia. Microglia express numerous receptors, including complement receptors, formyl peptide, NOD-type proteins, scavenger receptor A, Toll-like receptors, and TREM2, which play a key role in the immune response and neurodegeneration [99,100]. Also noteworthy is the NLR family pyrin domain containing 3 (NLRP3) inflammasome, whose activation leads to the conversion of pro-IL-1β and pro-IL-18, among others, to their active forms, enhancing inflammation within the microglia [101]. TREM2 is a surface receptor found on glial cells that, through interaction with the adaptor protein DAP12 (encoded by TYROBP), activates signaling pathways involved in chemotaxis, phagocytosis, survival, and proliferation of these cells. Its role in microglia includes the regulation of phagocytic motility and neuronal homeostasis. TREM2 mutations are associated with an increased risk of Alzheimer’s disease and other neurodegenerative conditions. The protein is involved in the removal of β-amyloid aggregates and tissue debris, which is crucial for protecting the brain. However, its dysregulation can exacerbate neuroinflammation, which poses a challenge for future possible therapeutic strategies [93,102,103].

The pathological feature that distinguishes Alzheimer’s disease and more than two dozen other neurodegenerative diseases is the abnormal folding and aggregation of the tau protein [104]. The microtubule-associated protein tau, among other things, plays a key role in maintaining the structure and function of neurons in the central nervous system. Accumulation of tau aggregates in cells is a hallmark of Alzheimer’s disease pathology. For many years it was assumed that changes in Aβ initiate the disease, leading to tau pathology and neurodegeneration, assuming that Aβ and tau act independently. However, a growing body of evidence points to a synergistic effect of the two pathologies, suggesting their interaction in the progression of Alzheimer’s disease [105,106,107]. Over the past two decades, the scientific evidence suggests that neuroinflammatory processes in the central nervous system play a key role in the pathogenesis of AD, becoming one of the main hallmarks of this pathology. Chronic inflammation combines changes in Aβ plaques and neurofibrillary degeneration associated with the tau protein, which are characteristic markers for the disease. Studies indicate that microglia activation progresses with age, and its severity correlates with the onset of Aβ accumulation and tau hyperphosphorylation. In an experiment on a mouse model, we investigated whether microglia activation enhances these processes. For this purpose, mice with induced Alzheimer’s disease were exposed to an inflammatory trigger—lipopolysaccharide. Although the processing of amyloid precursor protein was not disrupted, LPS significantly increased tau hyperphosphorylation, which was mediated by activation of cyclin-dependent kinase 5 and an increase in the p25 fragment [108,109]. In a mouse model of tauopathy, microglia activation and changes in synaptic structure have been found to be among the first signs of neurodegeneration. The authors suggested that activated microglia contribute to the severity of tau pathology by damaging dendrites and axons. The study also showed that inhibition of the inflammatory response can alleviate the effects of tau pathology [110]. Another study in a mouse model showed that a decrease in IL-10 activates microglia cells, leading to an increase in IL-6 levels and inducing hyperphosphorylation of the tau protein on epitopes key to Alzheimer’s disease in response to acute systemic inflammation [111].

A complex interaction between microglia cells, Aβ, tau protein, neurons, and inflammatory processes contributes to the development of AD. Modulation of this inflammatory response may represent a promising therapeutic target for the treatment of AD.

### 4.3. Treatment

Treatment of Alzheimer’s disease currently relies mainly on symptomatic treatment and causal methods of treating the disease are not currently widely available [78]. In June 2021, the Food and Drug Administration (FDA) approved aducanumab, which is an IgG1 monoclonal antibody, as a new treatment for Alzheimer’s disease targeting amyloid β, considered a key pathogenetic factor in the disease. Two years later, the FDA approved lecanemab, which in two clinical trials showed the ability to reduce amyloid in the brain and slow cognitive decline. Despite the promising results, the limited efficacy of these drugs indicates the need for further development of this group of therapies [112,113].

Current symptomatic treatments include only two classes of approved medications for the treatment of Alzheimer’s disease; *i*/inhibitors of the enzyme cholinesterase, i.e., donepezil, rivastigmine, or galantamine, and *ii*/N-methyl-D-aspartate antagonists, i.e., memantine, which are only effective in treating the symptoms of the disease but do not cure or prevent the disease [88,114].

Inhibition of acetylcholinesterase by inhibitors was the first pharmacological therapeutic target that showed efficacy in improving cognition, behavior, and the ability to perform daily activities in the treatment of Alzheimer’s disease. Donepezil, a drug from this class, is widely used in the treatment of Alzheimer’s disease. Studies have shown that eight months of treatment of mice with Alzheimer’s disease with donepezil led to improvements in neuroinflammatory changes, tau pathology, synaptic and neuronal loss, and reduced tau protein insolubility and phosphorylation [115,116]. Donepezil also inhibits the activation of AKT/MAPK signaling pathways, the NLRP3 inflammasome, and phosphorylation of transcription factors NF-kB and STAT3, which led to a reduction in LPS-induced neuroinflammatory responses in vitro in mice [117]. Interestingly, donepezil, in addition to its effects on the central nervous system in Alzheimer’s disease, also reduces inflammation and cell apoptosis in ulcerative colitis [118]. It was also found that administration of tacrine, rivastigmine, and donepezil to mice significantly attenuated lipopolysaccharide-induced increased IL-2 levels along with significant reductions in acetylcholinesterase activity [119].

Butyrylcholinesterase and neuroinflammation represent promising therapeutic targets for Alzheimer’s disease (AD). Pyranone-carbamate derivatives show selective inhibition of and anti-neuroinflammatory activity comparable to hydrocortisone. In a scopolamine-induced mouse model, these compounds alleviated cognitive and memory deficits, achieving efficacy similar to rivastigmine [120,121].

**Figure 3:**
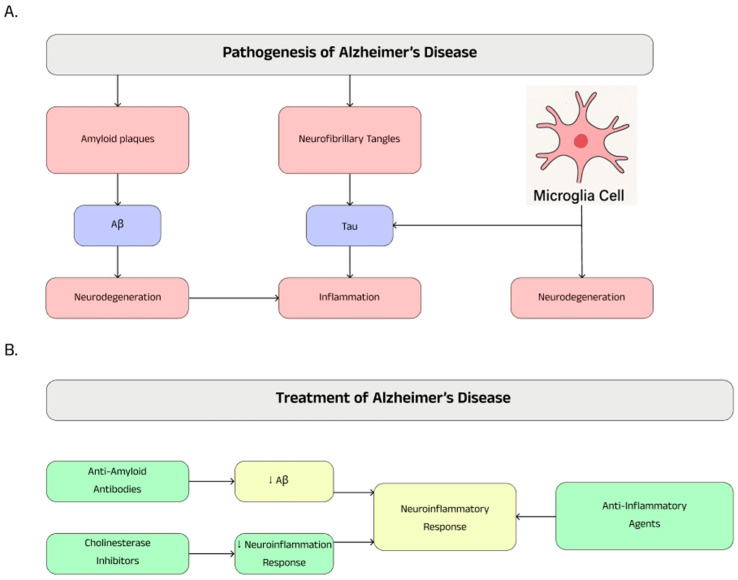
Pathogenesis (**A**) and therapeutic possibilities (**B**) in Alzheimer’s disease.

The influence of neuroinflammation on the pathogenesis and course of Alzheimer’s disease makes non-steroidal anti-inflammatory drugs a possible therapeutic treatment for preventing and slowing down the course of the disease. Diclofenac significantly reduced the incidence of Alzheimer’s disease compared to other NSAIDs such as ibuprofen, celecoxib, and naproxen in a large retrospective cohort study [122]. Another analysis of contemporary evidence confirms that the use of non-steroidal anti-inflammatory drugs may be significantly associated with a reduced risk of Alzheimer’s disease [123]. Cytokines such as TNF-α and IL-6 stimulate the expression of NFκB, leading to the production of Aβ in the brains of patients with Alzheimer’s disease. Drugs like celecoxib and rofecoxib, selective cyclooxygenase-2 (COX-2) inhibitors, as well as the non-selective COX inhibitor, indomethacin, demonstrate properties that alleviate neuroinflammatory responses by reducing the release of inflammatory mediators from microglia [124]. Summary information on Alzheimer’s disease is presented in Figure 3.

## 5. Inflammation in Multiple Sclerosis

### 5.1. Background Information

Multiple sclerosis (MS) is a chronic inflammatory disease in which myelin sheaths in the central nervous system are damaged. The process results in focal changes in the gray and white matter of the brain, as well as diffuse damage to neurons throughout the nervous system [125]. It is an autoimmune disease that leads to demyelination and nerve damage in the central nervous system. It is the leading cause of neurological disability in young adults, unrelated to injury [126]. The disease affects approximately 2.3 million individuals globally. It is most frequently diagnosed in individuals between the ages of 20 and 50, with a higher prevalence in women compared to men [127]. The diagnosis of multiple sclerosis is based on clinical evaluation and tests such as magnetic resonance imaging and lumbar puncture as there is no single diagnostic test. MS most often occurs in young adults, especially women, and manifests itself with recurrent neurological attacks. Early diagnosis, made possible by new criteria, allows for more effective treatment. Initially, MS changes are microscopic, and over time lead to symptoms such as sensory disturbances, muscle dysfunction, vision loss, and cognitive problems [128,129].

### 5.2. Pathogenesis

**Figure 4:**
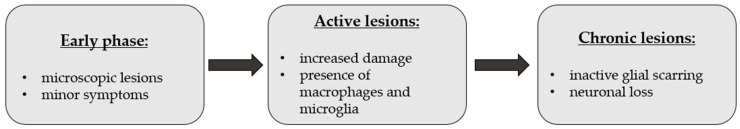
Progression of lesions in multiple sclerosis.

Multiple sclerosis is classified as an autoimmune disease in which the immune system plays a key role. Although the pathogenesis of MS was initially thought to be related to T lymphocytes, modern research points to the predominant role of B lymphocytes in the development of the disease. In the early stages of the disease, lesions in the central nervous system, known as active lesions, include loss of myelin, immune cell infiltration, blood–brain barrier damage, gliosis, and axonal damage. These lesions are characteristic of the relapsing–remitting form. Inactive lesions, on the other hand, are older demyelinating lesions with minimal glial and immune cell activity, which become more common in patients with a long-term course of the disease [129,130,131]. Active lesions are characterized by the presence of macrophages in the center, while astrocytosis also appears. The edge of these lesions is populated by microglia, which attract other inflammatory cells, as well as macrophages phagocytosing myelin. In the case of mixed active and inactive lesions, no significant number of astrocytes is observed, while microglia are clearly formed around the edge of the lesions [132]. Th lymphocytes are a subgroup of T cells that play an important role in initiating the immune response. This group includes Th17 lymphocytes, which secrete interleukin-17 (IL-17), a potent pro-inflammatory mediator that can lead to autoimmunity. Th17 cells are crucial in the immune processes involved in the pathogenesis of multiple sclerosis. Their interaction with the blood–brain barrier triggers pro-neuroinflammatory responses that contribute to the development of the disease. In addition, the interaction of Th17 with neurons and glial cells in the central nervous system enhances inflammatory processes, which play an important role in the course of MS [133,134]. In multiple sclerosis, the pathogenesis of the disease involves inflammation, which plays a key role in damage to myelin and neurons. Although anti-inflammatory drugs are not the mainstay of MS treatment, they can be used in some cases to alleviate the symptoms of inflammation and to treat relapses. Summary information on progression of lesions in multiple sclerosis is presented in Figure 4.

### 5.3. Treatment

Successful treatment of multiple sclerosis requires a comprehensive approach that takes into account controlling acute attacks, managing progressive deterioration, and alleviating symptoms that impede daily functioning. Therapeutic decisions should be made on the basis of the patient’s individual disease progression and functional, clinical, and imaging parameters, not solely on the basis of the clinical phenotype [126,135]. A characteristic of multiple sclerosis is the occurrence of flare-ups. This is what we call a relapse or exacerbation of the disease.

Disease-modifying therapies (DMTs) in multiple sclerosis aim to reduce disease activity and slow its progression. One such therapy is glatiramer acetate, which is approved for the treatment of MS. Studies have shown that glatiramer acetate can alter the balance of T lymphocytes, shifting the dominant pro-inflammatory Th1 phenotype to a more anti-inflammatory phenotype. Furthermore, it has been demonstrated that glatiramer acetate, in a dose-dependent manner, reduces the production of interleukin-17 (IL-17) and interferon-gamma (IFN-γ) by stimulating CD4+ T cells [136,137].

Treatment with ocrelizumab is classified as a highly effective disease-modifying therapy, ocrelizumab itself is a humanized anti-CD20 monoclonal antibody. It is administered intravenously. It is approved for the treatment of adult patients with relapsing forms of multiple sclerosis and primary progressive multiple sclerosis. The efficacy of ocrelizumab in reducing the relapse rate and disease activity in multiple sclerosis patients has been confirmed in pivotal trials comparing it with interferon β-1a [138,139].

## 6. Methods

The selection of the literature was made on the basis of a review of the literature in the fields of medicine, pharmacy, and biology. In this review, research articles and review articles were assessed after electronic searches conducted between September 2024 and March 2025 in the PubMed and Google Scholar databases. The search in the general part of this article was performed based on the following keywords: neurodegenerative diseases, microglial cells, neuroinflammation, astrocytes. In the case of individual diseases, the keywords were a combination of the name of a given disease entity with the following phrases: pathogenesis, treatment. In order to discuss a given issue in detail, keywords were used that contained in their name the names of factors or characteristics of a given disease or international names of medicinal substances. The literature used comes mostly from the last five years but in order to supplement the topic, older literature data were also used.

## 7. Conclusions

In summary, the presented article describes and focuses on the most important information regarding neurodegenerative diseases in the context of inflammation. The first part draws attention to the growing problem of the increasing incidence of neurodegenerative diseases. The most important general issues regarding neurodegenerative diseases are discussed, including their causes, and the phenomenon of neuroinflammation is discussed. The next part presents the participation of individual cells of the central nervous system such as microglial cells and astrocytes in the course of the discussed pathological conditions. The following main part of this article pays special attention to the role of the discussed phenomenon of inflammation in the pathogenesis and treatment of individual neurodegenerative diseases such as Parkinson’s disease, Alzheimer’s disease, and multiple sclerosis.

## 8. Future Directions

Future research in neurodegenerative diseases is increasingly focused on understanding the role of inflammation as an important factor in the pathogenesis of CNS diseases, and, in addition, chronic inflammatory processes in the nervous system that may contribute to the progression of Alzheimer’s diseases, Parkinson’s diseases, or multiple sclerosis through activation of the microglia and astrocytes and the release of pro-inflammatory cytokines. Potential therapeutic strategies include the development of drugs that modulate the inflammatory process, such as cytokine inhibitors, microglia modulators, or agents that inhibit inflammasome activation. Moreover, therapies targeting key molecular pathways involved in the inflammation process are receiving increasing attention, which may make it possible to slow or inhibit the progression of neurodegeneration. In the future, studies on developing biomarkers that allow for the early detection of inflammation, facilitating the implementation of therapies at a stage when they are still most effective, will also be important. The integration of immunomodulatory approaches with innovative methods such as gene or cell therapy represents a promising path forward in the treatment of neurodegenerative diseases.

## Data Availability

Data sharing is not applicable.

## Abbreviations

The following abbreviations are used in this manuscript:

Aβ	Amyloid β
AD	Alzheimer’s disease
BBB	Blood–brain barrier
CNS	Central nervous system
COX	Cyclooxygenase
DMTs	Disease-modifying therapies
FDA	Food and Drugs Administration
IFN	Interferon
IL	Interleukin
NF-κB	Nuclear factor kappa-light-chain-enhancer of activated B cells
MS	Multiple sclerosis
NO	Nitric oxide
NSAIDs	Non-steroidal anti-inflammatory drugs
PD	Parkinson’s disease
SCFA	Short-chain fatty acid
TNF-α	Tumor necrosis factor-α
TREM2	Triggering receptor expressed on myeloid cell 2
WHO	World Health Organization
